# Lifestyle, medication use, and age considerations with acne vulgaris: A prospective study

**DOI:** 10.1002/jvc2.521

**Published:** 2024-08-15

**Authors:** Ashley M. Snyder, Caroline J. Stone, Nicole Ufkes, Tom Greene, Mary C. Playdon, Maureen A. Murtaugh, Megan E. Vanneman, Aaron M. Secrest

**Affiliations:** ^1^ Department of Dermatology University of Utah Salt Lake City Utah USA; ^2^ Department of Population Health Sciences University of Utah Salt Lake City Utah USA; ^3^ Division of Epidemiology, Department of Internal Medicine University of Utah Salt Lake City Utah USA; ^4^ School of Medicine University of Utah Salt Lake City Utah USA; ^5^ Department of Nutrition & Integrative Physiology University of Utah Salt Lake City Utah USA; ^6^ Cancer Control and Population Sciences, Huntsman Cancer Institute University of Utah Salt Lake City Utah USA; ^7^ Department of Dermatology Christchurch Hospital, Health New Zealand Te Whatu Ora Christchurch New Zealand

**Keywords:** acne, disease control, diet, epidemiology, mental health, quality of life

## Abstract

**Background:**

Lifestyle has been associated with acne, but few studies assess how the relationship changes over time. Observational studies often overlook the effects of acne medication use and participant age in relationships with lifestyle‐related factors.

**Objectives:**

To describe relationships between lifestyle‐related factors, medication use, and age in adolescent and young adult acne patients and acne‐free controls.

**Methods:**

This prospective study recruited 12‐ to 24‐year‐olds with or without acne at baseline. Surveys were electronically administered at enrolment and again 6 weeks later. Analyses were conducted on all participants who had complete baseline data (*N* = 190) and participants who had complete baseline and follow‐up data (*N* = 61).

**Results:**

Among 190 participants who completed the baseline survey, ages ranged from 12 to 24 years, but acne cases were concentrated in the middle of this range while controls had comparably more participants with ages towards the extremes. Among 61 participants who completed both baseline and follow‐up surveys, no participants indicated worse acne over the 6 weeks, and most acne cases believed their acne improved (*n* = 25 [69.4%]). Acne cases who used medication daily (*N* = 24) saw improved emotion‐related quality of life between the two assessments (mean ± standard deviation: 43.4 ± 24.4 to 29.1 ± 23.7; *p* < 0.001). Among acne cases who used medication daily, average fruit or vegetable consumption increased from 2.4 ± 2.0 to 3.0 ± 2.9 times per day over the preceding 7 days (*p* = 0.02). Among acne cases who believed their acne improved over the 6 weeks post‐dermatology visit (*N* = 25), average days of skin picking over the previous 7 days declined between assessments (3.9 ± 2.4 to 2.4 ± 1.9 days; *p* = 0.003).

**Conclusions:**

Medication use and age differences should be considered when designing future studies on acne and lifestyle‐related factors.

## INTRODUCTION

Lifestyle medicine holds promising complementary treatment options for acne vulgaris. Observational studies support the connection between acne and diet, with negative associations between acne with intake of fish, fruits, vegetables, and following a Mediterranean diet and positive associations with dairy intake.^1^, ^2^, ^3^, ^4^, ^5^ Clinical trials show that a low glycemic load diet can reduce numbers of acne lesions.^6^, ^7^, ^8^, ^9^, ^10^, ^11^, ^12^, ^13^ Observational studies have also shown associations between acne with stress, depression, anxiety, anger and sleep disturbance,^14^, ^15^, ^16^ and a small randomised controlled trial indicated that stress management could improve acne.^17^ Ethnographic/population studies have shown that specific populations consuming traditional diets rarely experienced acne, which became more prominent as dietary patterns became Westernised.^18^, ^19^, ^20^, ^21^, ^22^ However, the number of lifestyle‐related factors that could differ between populations makes the connection between acne and lifestyle complicated. For example, mental and emotional health in people with acne might also be related to smoking,^23^, ^24^ physical activity,^25^, ^26^, ^27^ skin picking^28^ and use of make‐up.^29^

Medication use and age are important potential factors to consider when studying acne and lifestyle. Hypothetically, health consciousness associated with a patient caring more about diet and mental health might also be associated with consistent use of acne medications. Further, a patient whose acne permanently resolves after an isotretinoin course will no longer have acne, regardless of lifestyle. Some observational studies on diet and acne account for general or specific medication use,^30^, ^31^, ^32^, ^33^, ^34^, ^35^ but others do not account for medications.^25^, ^36^, ^37^ This is problematic because patients are often written prescriptions or educated on over‐the‐counter topicals. Age should also be a consideration. Adult acne (i.e., acne persisting past 25 years old^38^) has a different pathogenesis and thus different risk. Some studies on dietary factors and acne have included participants over age 25, with one study including individuals up to age 77 years.^38^, ^39^, ^40^ Similarly, participants who are too young to develop acne might not be appropriate controls. This is also problematic for lifestyle studies if lifestyle choices differ by age. For example, purchasing alcohol is restricted to individuals aged 21 years or older in the state of Utah.^41^ Thus, restricting inclusion in a study to an appropriate age range for the acne category or lifestyle factor of interest may be necessary.

This exploratory prospective study aimed to (1) assess associations between adolescent acne with lifestyle‐related factors, including diet and mental health; (2) explore how age is distributed in acne cases versus acne‐free controls and how age relates to lifestyle factors, such as alcohol; and (3) explore how focusing on participants with more frequent (e.g., daily) acne medication use affects average frequency or severity of lifestyle factors 6 weeks after a dermatology visit. The overarching goal of this project was to provide evidence for researchers to consider when designing future research on acne and lifestyle.

## MATERIALS AND METHODS

This project received ethics approval from the University of Utah Institutional Review Board (#140574). Participants were emailed a baseline REDCap^42^, ^43^ survey on the day of their dermatology visit and a follow‐up survey 6 weeks later to allow time for new acne medications to take effect. Both surveys expired within 24 h after being sent to assess exposures primarily before dermatologic assessment (for baseline) and within the 6 weeks after dermatologic assessment. Surveys asked about demographics (assessed with original and modified questions from the Standard High School version of the 2021 State and Local Youth Risk Behavior Survey [YRBS]), diet (original and modified YRBS questions), mental and emotional health (NIH Toolbox Perceived Stress Fixed Form Age 18 + v2.0, PROMIS Short Form v1.0 ‐ Depression 4a, PROMIS Short Form v1.0 ‐ Anxiety 4a, PROMIS Short Form v1.1 ‐ Anger 5a, and PROMIS Short Form v1.0 ‐ Sleep Disturbance 4a, and 25‐item Connor‐Davidson Resilience Scale), skin‐related quality of life (SRQL; Skindex‐16 with modified instructions), physical activity (YRBS), cigarette smoking (modified YRBS question), skin irritants (investigator‐developed), and medications (investigator‐developed; Supporting Information: Material 1 and Supporting Information: Table 1).

### Participants and setting

Recruitment occurred from late November 2021 to mid‐August 2022. Participants were between the ages of 12 to 24 years, and most were recruited from three clinic locations of the University of Utah Department of Dermatology. An additional convenience sample was recruited for controls only, due to the difficulty of finding 12‐ to 24‐year‐old dermatology patients who did not have any acne. Convenience sample participants included family, friends, or acquaintances of recruiters and did not have to be physically located in Utah to participate. Non‐clinician recruiters were allowed to assess convenience sample participants' facial acne status. Dermatology visits could be done in‐person or via online video. For convenience sample controls, the enrolment meeting counted as a 'dermatology visit' for study purposes.

An Investigator's Global Assessment (IGA), with scores ranging from 0 (clear) to 4 (severe), was used to evaluate acne severity.^44^, ^45^ Some clinicians used a modified IGA scoring system that included an additional higher severity category, but if assigned, this category was combined with the 'severe' category in the primary IGA scoring system for consistency (Supporting Information: Table 2). For simplicity, cases were defined as any acne (scores 1 to 4) and controls as no acne (score of 0). Acne status was determined with the help of clinicians—including board‐certified dermatologists, dermatology residents, and advanced practice clinicians—who referred patients to the study. Both acne and non‐acne patients could potentially have other skin conditions, so information on other dermatologic diagnoses from enrolment clinic visits was also collected from electronic medical records.

Female participants were excluded if they were currently pregnant or breastfeeding or had ever been diagnosed with polycystic ovary syndrome since polycystic ovary syndrome may involve acne but has a unique pathophysiology. Controls were excluded if they had a chronic inflammatory skin disease flaring at their dermatology visit or if they had ever used isotretinoin.

### Sample size

Sample size was determined for the baseline survey to provide adequate power for a separate analysis that evaluated stress as a continuous exposure variable and acne presence as a binary yes/no outcome variable. A 2:1 ratio of cases:controls was used to provide adequate power to detect a 1.6^46^ fold increase in the odds of acne per 1 standard deviation increase in stress, allowing for an *R* ^2^ of up to 0.15 between the exposure and covariates.^47^ The resulting target sample size was 189 participants with complete data (126 cases and 63 controls). Power Analysis & Sample Size (PASS) software (NCSS) was used for sample size calculations.

### Statistical analyses

Only complete surveys were used. Descriptive statistics were calculated, with tests including Fisher's exact test for relationships between categorical variables, Student's *t*‐test for comparing mean levels of non‐paired continuous variables between two groups, paired *t*‐test for comparing means of continuous variables between the baseline and follow‐up surveys, and one‐way analysis of variance for comparing means of non‐paired continuous variables between more than two categories. Spearman correlations were also used for assessing relationships between continuous variables. An *α* of 0.05 was used as the cutoff for statistical significance. Stata version 16 software (StataCorp) was used for analyses. Daily medication use was explored in detail only for cases since so few controls indicated daily medication use.

## RESULTS

A total of 328 participants consented to the study, and 190 individuals (57.9%) completed the baseline survey in its entirety and had all information necessary for calculations; only 61 of these 190 participants also completed a follow‐up survey (32.1%). Three acne cases completed the follow‐up but not baseline and were excluded from analyses.

Among the 190 participants with complete baseline surveys, there were 127 acne cases and 63 acne‐free controls (for demographics and medication use, see Table 1). Most acne cases used a medication for acne. Notably, a nontrivial minority of acne‐free controls were using acne treatments. Most participants (*n* = 112 [58.9%]) were somewhat or very satisfied with their treatment for acne or for another skin condition if they did not have acne. Only two participants smoked cigarettes, so this variable was excluded from further analyses.

**Table 1:** Baseline demographics and medication use by acne status and severity among 190 adolescents and young adults with completed baseline surveys.

	Binary acne status (*N* = 190)			Acne severity in cases (*N* = 127)				
	Acne cases *N* = 127	Acne‐free controls *N* = 63	*p*‐Value	Severe *N* = 7	Moderate *N* = 46	Mild *N* = 41	Almost clear *N* = 33	*p*‐Value
Age, mean ± SD	18.0 ± 3.4	20.1 ± 3.9	**<0.001**	19.0 ± 2.8	17.4 ± 3.1	18.1 ± 3.5	18.7 ± 3.8	0.36
Female, *n* (%)	82 (64.6)	38 (60.3)	0.63	4 (57.1)	31 (67.4)	25 (61.0)	22 (66.7)	0.87
Non‐Hispanic White, *n* (%)	96 (75.6)	58 (92.1)	**0.006**	2 (28.6)	37 (80.4)	33 (80.5)	24 (72.7)	**0.04**
Current education, *n* (%)			**0.002**					0.55
Middle school	14 (11.0)	9 (14.3)		0 (0.0)	6 (13.0)	6 (14.6)	2 (6.1)	
High school	46 (36.2)	9 (14.3)		2 (28.6)	20 (43.5)	12 (29.3)	12 (36.4)	
College or trade school after high school	50 (39.4)	24 (38.1)		4 (57.1)	17 (37.0)	18 (43.9)	11 (33.3)	
Graduate school	4 (3.1)	7 (11.1)		0 (0.0)	0 (0.0)	2 (4.9)	2 (6.1)	
No longer in school (graduated or GED)	13 (10.2)	14 (22.2)		1 (14.3)	3 (6.5)	3 (7.3)	6 (18.2)	
Current job(s), *n* (%)			**0.03**					0.26
Full‐time job only	23 (18.1)	23 (36.5)		0 (0.0)	5 (10.9)	7 (17.1)	11 (33.3)	
Part‐time job(s) only	50 (39.4)	17 (27.0)		4 (57.1)	19 (41.3)	15 (36.6)	12 (36.4)	
Both full‐ and part‐time jobs	1 (0.8)	1 (1.6)		0 (0.0)	1 (2.2)	0 (0.0)	0 (0.0)	
Unemployed	53 (41.7)	22 (34.9)		3 (42.9)	21 (45.7)	19 (46.3)	10 (30.3)	
Any acne medication, *n* (%)	92 (72.4)	11 (17.5)	**<0.001**	3 (42.9)	34 (73.9)	35 (85.4)	20 (60.6)	**0.03**
Topical acne medication, *n* (%)	66 (52.0)	9 (14.3)	**<0.001**	3 (42.9)	26 (56.5)	23 (56.1)	14 (42.4)	0.55
Systemic acne medication, *n* (%)	49 (38.6)	6 (9.5)	**<0.001**	1 (14.3)	15 (32.6)	22 (53.7)	11 (33.3)	0.09

Abbreviation: SD, standard deviation.

Baseline demographic data for the 61 who completed both surveys (36 cases and 25 controls) are shown in Table 2.

**Table 2:** Baseline demographics by acne status and severity for 61 adolescents and young adults who completed both surveys.

	Binary acne status (*N* = 61)			Acne severity in cases (*N* = 36)				
	Acne cases *N* = 36	Acne‐free controls *N* = 25	*p*‐Value	Severe *N* = 3	Moderate *N* = 9	Mild *N* = 15	Almost clear *N* = 9	*p*‐Value
Age, mean ± SD	19.2 ± 3.1	20.8 ± 3.4	0.06	18.0 ± 1.0	18.7 ± 3.6	19.3 ± 2.3	20.0 ± 4.3	0.74
Female, *n* (%)	25 (69.4)	18 (72.0)	>0.999	1 (33.3)	7 (77.8)	10 (66.7)	7 (77.8)	0.50
Non‐Hispanic White, *n* (%)	29 (80.6)	22 (88.0)	0.50	1 (33.3)	9 (100.0)	12 (80.0)	7 (77.8)	0.09
Current education, *n* (%)			0.09					0.10
Middle school	2 (5.6)	3 (12.0)		0 (0.0)	1 (11.1)	0 (0.0)	1 (11.1)	
High school	8 (22.2)	1 (4.0)		1 (33.3)	2 (22.2)	3 (20.0)	2 (22.2)	
College or trade school after high school	18 (50.0)	10 (40.0)		1 (33.3)	4 (44.4)	11 (73.3)	2 (22.2)	
Graduate school	1 (2.8)	4 (16.0)		0 (0.0)	0 (0.0)	1 (6.7)	0 (0.0)	
No longer in school (graduated or GED)	7 (19.4)	7 (28.0)		1 (33.3)	2 (22.2)	0 (0.0)	4 (44.4)	
Current job(s), *n* (%)			0.52					0.10
Full‐time job only	9 (25.0)	9 (36.0)		0 (0.0)	2 (22.2)	2 (13.3)	5 (55.6)	
Part‐time job(s) only	14 (38.9)	8 (32.0)		0 (0.0)	3 (33.3)	8 (53.3)	3 (33.3)	
Both full‐ and part‐time jobs	0 (0.0)	1 (4.0)		0 (0.0)	0 (0.0)	0 (0.0)	0 (0.0)	
Unemployed	13 (36.1)	7 (28.0)		3 (100.0)	4 (44.4)	5 (33.3)	1 (11.1)	
Any acne medication, *n* (%)	24 (66.7)	2 (8.0)	**<0.001**	2 (66.7)	6 (66.7)	11 (73.3)	5 (55.6)	0.88
Topical acne medication, *n* (%)	17 (47.2)	2 (8.0)	**0.002**	2 (66.7)	6 (66.7)	7 (46.7)	2 (22.2)	0.28
Systemic acne medication, *n* (%)	12 (33.3)	2 (8.0)	**0.03**	0 (0.0)	1 (11.1)	8 (53.3)	3 (33.3)	0.13

Abbreviation: SD, standard deviation.

### Differences in lifestyle‐related factors by age at baseline

**Figure 1:**
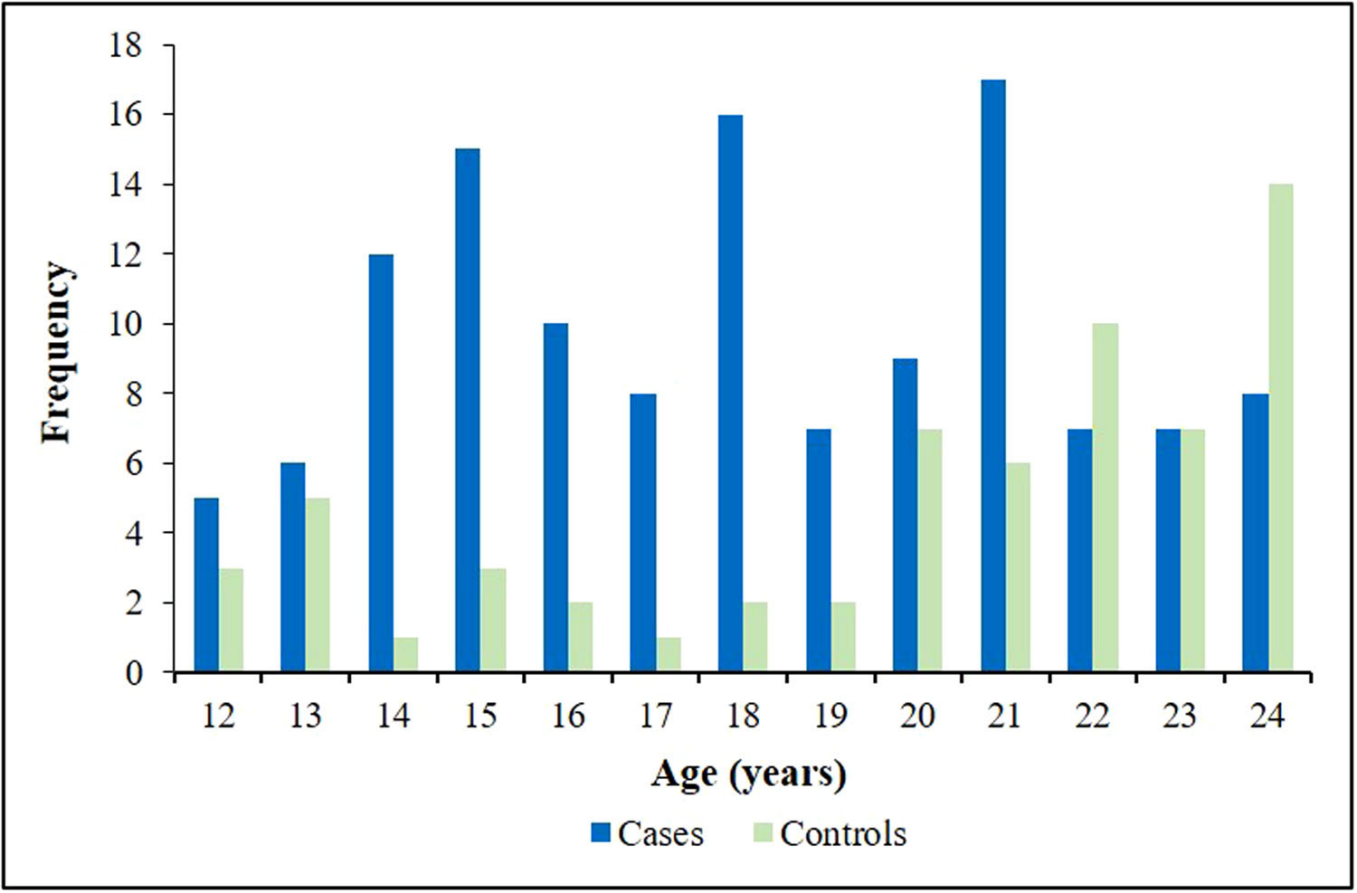
Age distribution for acne cases and acne‐free controls (*N* = 190).

Participant age at baseline ranged from 12 to 24 years. Acne cases were more common in the middle of this range, while acne‐free controls were more common on the younger and older ends of the range (Figure 1).

Lifestyle‐related factors at baseline differed by age for cases and controls (Table 3). Fruit juice consumption was more frequent at younger ages among controls. Soda and milk consumption were higher at younger ages for both cases and controls. Alcohol consumption was greater among older‐aged cases. Days of breakfast consumption was greater among younger‐aged controls. Depression was worse with older age among cases. Physical activity was higher among younger cases. An ad hoc assessment of alcohol with mental and emotional health factors was conducted but all correlations were weak (Table 4).

**Table 3:** Spearman correlations between age and lifestyle‐related factors at baseline by case or control status (*N* = 190).

Lifestyle‐related factor	Acne cases *N* = 127		Acne‐free controls *N* = 63	
	Rho	*p*‐Value	Rho	*p*‐Value
Fruit juice	0.05	0.58	−0.34	**0.006**
Fruits or vegetables	0.11	0.21	0.01	0.96
Soda	−0.27	**0.002**	−0.34	**0.006**
Milk	−0.28	**0.002**	−0.31	**0.01**
Alcohol	0.45	**<0.001**	0.21	0.10
Breakfast	0.16	0.07	−0.31	**0.01**
Stress	0.15	0.09	0.16	0.21
Depression	0.23	**0.008**	0.11	0.38
Anxiety	0.11	0.19	0.05	0.71
Anger	0.10	0.28	−0.08	0.55
Sleep	0.12	0.18	0.09	0.47
Emotional resilience	−0.03	0.73	−0.22	0.08
Physical activity	−0.22	**0.01**	−0.13	0.30

**Table 4:** Spearman correlations between alcohol and mental and emotional health factors at baseline by case or control status (*N* = 190).

Lifestyle‐related factor	Acne cases *N* = 127		Acne‐free controls *N* = 63	
	Rho	*p*‐Value	Rho	*p*‐Value
Stress	0.05	0.58	0.20	0.12
Depression	0.11	0.23	0.13	0.33
Anxiety	0.10	0.25	0.10	0.41
Anger	0.04	0.64	0.04	0.77
Sleep	0.02	0.78	0.21	0.11
Emotional resilience	<0.001	>0.99	−0.22	0.08

### Changes in medication use over time

Among the 61 participants who completed both baseline and 6‐week follow‐up surveys, no participants indicated worse acne over the 6 weeks, and most acne cases believed their acne improved (Table 5). Some participants who were classified as acne‐free controls at baseline indicated acne improvement. All female participants reported having a menstrual period, indicating that they had started puberty and thus might be susceptible to acne.

**Table 5:** Medication (med) adherence at 6‐week follow‐up (*N* = 61).

	Binary acne status (*N* = 61)			Acne severity in cases (*N* = 36)				
	Acne cases *N* = 36	Acne‐free controls *N* = 25	*p*‐Value	Severe *N* = 3	Moderate *N* = 9	Mild *N* = 15	Almost clear *N* = 9	*p*‐Value
Received med at baseline visit, *n* (%)	34 (94.4)	7 (28.0)	**<0.001**	3 (100.0)	9 (100.0)	15 (100.0)	7 (77.8)	0.20
Average use of med received, *n* (%)*			0.07					0.33
Never	1 (2.9)	0 (0.0)		0 (0.0)	0 (0.0)	0 (0.0)	1 (14.3)	
Less than once a week	0 (0.0)	2 (28.6)		0 (0.0)	0 (0.0)	0 (0.0)	0 (0.0)	
Once a week	1 (2.9)	0 (0.0)		0 (0.0)	0 (0.0)	0 (0.0)	1 (14.3)	
Between 2 to 7 days each week	8 (23.5)	1 (14.3)		1 (33.3)	3 (33.3)	4 (26.7)	0 (0.0)	
Every day	24 (70.6)	4 (57.1)		2 (66.7)	6 (66.7)	11 (73.3)	5 (71.4)	
Satisfaction with med received, *n* (%)*			0.75					0.89
Very dissatisfied	1 (3.0)	0 (0.0)		0 (0.0)	0 (0.0)	1 (6.7)	0 (0.0)	
Somewhat dissatisfied	2 (6.1)	0 (0.0)		0 (0.0)	1 (11.1)	0 (0.0)	1 (16.7)	
Neither satisfied nor dissatisfied	5 (15.2)	1 (14.3)		0 (0.0)	1 (11.1)	3 (20.0)	1 (16.7)	
Somewhat satisfied	15 (45.5)	2 (28.6)		2 (66.7)	5 (55.6)	5 (33.3)	3 (50.0)	
Very satisfied	10 (30.3)	4 (57.1)		1 (33.3)	2 (22.2)	6 (40.0)	1 (16.7)	
Used other med since baseline visit, *n* (%)	5 (13.9)	3 (12.0)	>0.999	0 (0.0)	3 (33.3)	0 (0.0)	2 (22.2)	0.09
Average use of other med, *n* (%)*			0.79					>0.999
Less than once a week	1 (20.0)	0 (0.0)		0 (0.0)	0 (0.0)	0 (0.0)	1 (50.0)	
Once a week	1 (20.0)	1 (33.3)		0 (0.0)	1 (33.3)	0 (0.0)	0 (0.0)	
Between 2 to 7 days each week	0 (0.0)	1 (33.3)		0 (0.0)	0 (0.0)	0 (0.0)	0 (0.0)	
Every day	3 (60.0)	1 (33.3)		0 (0.0)	2 (66.7)	0 (0.0)	1 (50.0)	
Satisfaction with other med, *n* (%)*			>0.999					>0.999
Very dissatisfied	1 (20.0)	0 (0.0)		0 (0.0)	1 (33.3)	0 (0.0)	0 (0.0)	
Somewhat dissatisfied	1 (20.0)	0 (0.0)		0 (0.0)	0 (0.0)	0 (0.0)	1 (50.0)	
Neither satisfied nor dissatisfied	2 (40.0)	1 (33.3)		0 (0.0)	1 (33.3)	0 (0.0)	1 (50.0)	
Somewhat satisfied	0 (0.0)	1 (33.3)		0 (0.0)	0 (0.0)	0 (0.0)	0 (0.0)	
Very satisfied	1 (20.0)	1 (33.3)		0 (0.0)	1 (33.3)	0 (0.0)	0 (0.0)	
Acne severity since baseline visit, *n* (%)			**<0.001**					0.09
Not applicable	2 (5.6)	21 (84.0)		1 (33.3)	0 (0.0)	0 (0.0)	1 (11.1)	
Acne improved	25 (69.4)	3 (12.0)		1 (33.3)	7 (77.8)	13 (86.7)	4 (44.4)	
Acne stayed the same	9 (25.0)	1 (4.0)		1 (33.3)	2 (22.2)	2 (13.3)	4 (44.4)	
Satisfaction with treatment compared to baseline, *n* (%)			**0.01**					0.51
Much more dissatisfied	0 (0.0)	1 (4.0)		0 (0.0)	0 (0.0)	0 (0.0)	0 (0.0)	
Somewhat more dissatisfied	2 (5.6)	0 (0.0)		0 (0.0)	0 (0.0)	2 (13.3)	0 (0.0)	
Neither more satisfied nor dissatisfied	11 (30.6)	16 (64.0)		0 (0.0)	3 (33.3)	3 (20.0)	5 (55.6)	
Somewhat more satisfied	13 (36.1)	7 (28.0)		2 (66.7)	4 (44.4)	4 (26.7)	3 (33.3)	
Much more satisfied	10 (27.8)	1 (4.0)		1 (33.3)	2 (22.2)	6 (40.0)	1 (11.1)	

* Smaller sample size due to dependence on answers to a previous question.

Among 24 acne cases who indicated daily average use of medication received at baseline, the mean ( ± standard deviation) Skindex‐16 emotions score for SRQL decreased over time from 43.4 ( ± 24.4) to 29.1 ( ± 23.7), indicating improved emotion‐related quality of life (*p* < 0.001); conversely, significant change was not observed with the symptoms (20.0 ± 12.6 to 19.8 ± 18.3; *p* = 0.96) or functioning (12.8 ± 16.8 to 10.1 ± 20.1; *p* = 0.23) domains.

### Changes in skin irritants over time

Among the 61 participants who completed both surveys, the use of make‐up declined from 17 (47.2%) to 12 (33.3%) in acne cases but rose from 12 (48.0%) to 15 (60.0%) in controls over 6 weeks, though neither association was statistically significant (*p* = 0.34 and *p* = 0.57, respectively). The average number of days controls self‐reported picking their skin over the previous 7 days did not significantly change (baseline: 1.3 ± 1.7; follow‐up: 1.3 ± 1.8; *p* = 0.89); however, reported skin picking decreased from an average of 3.8 ( ± 2.4) to 2.8 ( ± 2.1) days among acne cases (*p* = 0.008). Among the 25 acne cases who indicated their acne improved over 6 weeks, the average number of days of skin‐picking decreased from 3.9 ( ± 2.4) to 2.4 ( ± 1.9) days (*p* = 0.003). Similarly, when specifically assessing the 24 acne cases who used their baseline prescribed medication daily, skin‐picking decreased between baseline (3.7 ± 2.4) and follow‐up (2.6 ± 2.0; *p* = 0.04).

### Changes in lifestyle‐related factors over time

Values were generally similar between baseline and follow‐up for lifestyle‐related factors, though anger scores improved among acne cases over the 6 weeks (Table 6). When comparing changes specifically among acne cases who used the baseline prescribed medication daily (Table 7), the difference in fruit or vegetable consumption between baseline and follow‐up increased compared to the broader sample that was not specific to daily medication use (Table 6). Additionally, values appeared similar when comparing cases and controls at both baseline and follow‐up, though sleep scores were worse in acne cases compared to controls (Table 8).

**Table 6:** Changes in mean ± SD of lifestyle‐related factors over 6 weeks by acne status (*N* = 61).

	Acne cases (*N* = 36)			Acne‐free controls (*N* = 25)		
Lifestyle‐related factor	Baseline	Follow‐up	*p*‐Value	Baseline	Follow‐up	*p*‐Value
Fruit juice	0.2 ± 0.5	0.2 ± 0.5	0.99	0.2 ± 0.5	0.2 ± 0.4	0.11
Fruits or vegetables	2.3 ± 1.7	2.7 ± 2.5	0.06	2.5 ± 1.2	2.4 ± 1.2	0.63
Soda	0.4 ± 0.6	0.3 ± 0.4	0.24	0.3 ± 0.4	0.2 ± 0.3	0.22
Milk	0.7 ± 0.8	0.6 ± 0.8	0.25	0.5 ± 0.7	0.3 ± 0.4	0.07
Alcohol	0.0 ± 0.1	0.0 ± 0.1	0.57	0.1 ± 0.2	0.1 ± 0.2	0.33
Breakfast	4.6 ± 2.4	4.6 ± 2.2	0.93	4.5 ± 2.3	4.4 ± 2.6	0.89
Stress	52.5 ± 10.0	50.9 ± 12.1	0.18	48.1 ± 11.3	48.7 ± 13.5	0.66
Depression	50.2 ± 9.3	51.5 ± 10.1	0.27	48.7 ± 9.4	50.8 ± 9.6	0.07
Anxiety	52.9 ± 9.1	53.0 ± 10.9	0.92	53.8 ± 8.9	52.2 ± 11.9	0.23
Anger	51.0 ± 7.8	48.4 ± 9.1	**0.009**	49.0 ± 9.0	48.9 ± 8.4	0.94
Sleep	53.3 ± 7.5	53.9 ± 8.2	0.62	44.9 ± 8.8	48.6 ± 8.0	0.05
Emotional resilience	66.8 ± 16.4	65.8 ± 17.7	0.45	71.7 ± 13.9	72.4 ± 15.1	0.60
Physical activity	3.5 ± 2.3	3.6 ± 2.4	0.78	3.0 ± 1.8	3.2 ± 2.1	0.35

Abbreviation: SD, standard deviation.

**Table 7:** Changes in mean ± SD of lifestyle‐related factors among acne cases who used prescribed medication daily on average over 6 weeks (*N* = 24).

Lifestyle‐related factor	Baseline	Follow‐up	*p*‐Value
Fruit juice	0.3 ± 0.6	0.3 ± 0.6	0.91
Fruits or vegetables	2.4 ± 2.0	3.0 ± 2.9	**0.02**
Soda	0.4 ± 0.6	0.2 ± 0.2	0.18
Milk	0.7 ± 0.9	0.7 ± 1.0	0.37
Alcohol	0.0 ± 0.1	0.0 ± 0.1	>0.999
Breakfast	4.9 ± 2.4	4.7 ± 2.0	0.59
Stress	51.6 ± 8.9	50.3 ± 12.5	0.40
Depression	50.1 ± 9.5	50.7 ± 10.4	0.71
Anxiety	53.1 ± 9.0	51.8 ± 11.7	0.32
Anger	50.9 ± 7.4	47.0 ± 8.6	**0.004**
Sleep	52.6 ± 7.3	52.1 ± 7.9	0.72
Emotional resilience	68.3 ± 16.8	67.0 ± 17.4	0.42
Physical activity	3.7 ± 2.6	3.7 ± 2.7	0.90

Abbreviation: SD, standard deviation.

**Table 8:** Changes in mean ± SD of lifestyle‐related factors by acne status at baseline and follow‐up (*N* = 61).

	Baseline			Follow‐up		
Lifestyle‐related factor	Acne cases *N* = 36	Controls *N* = 25	*p*‐Value	Acne cases *N* = 36	Controls *N* = 25	*p*‐Value
Fruit juice	0.2 ± 0.5	0.2 ± 0.5	0.97	0.2 ± 0.5	0.2 ± 0.4	0.50
Fruits or vegetables	2.3 ± 1.7	2.5 ± 1.2	0.61	2.7 ± 2.5	2.4 ± 1.2	0.56
Soda	0.4 ± 0.6	0.3 ± 0.4	0.63	0.3 ± 0.4	0.2 ± 0.3	0.77
Milk	0.7 ± 0.8	0.5 ± 0.7	0.37	0.6 ± 0.8	0.3 ± 0.4	0.15
Alcohol	0.0 ± 0.1	0.1 ± 0.2	0.11	0.0 ± 0.1	0.1 ± 0.2	0.30
Breakfast	4.6 ± 2.4	4.5 ± 2.3	0.83	4.6 ± 2.2	4.4 ± 2.6	0.75
Stress	52.5 ± 10.0	48.1 ± 11.3	0.11	50.9 ± 12.1	48.7 ± 13.5	0.52
Depression	50.2 ± 9.3	48.7 ± 9.4	0.54	51.4 ± 10.1	50.8 ± 9.6	0.78
Anxiety	52.9 ± 9.1	53.8 ± 8.9	0.70	53.0 ± 10.9	52.2 ± 11.9	0.78
Anger	51.0 ± 7.8	49.0 ± 9.0	0.37	48.4 ± 9.1	48.9 ± 8.4	0.82
Sleep	53.3 ± 7.5	44.9 ± 8.8	**<0.001**	53.9 ± 8.2	48.6 ± 8.0	**0.02**
Emotional resilience	66.8 ± 16.4	71.7 ± 13.9	0.23	65.8 ± 17.7	72.4 ± 15.1	0.13
Physical activity	3.5 ± 2.3	3.0 ± 1.8	0.40	3.6 ± 2.4	3.2 ± 2.1	0.60

Abbreviation: SD, standard deviation.

Since Utah's legal minimum age for alcohol purchase is 21 years,^41^ additional analyses with alcohol were conducted with data from participants who completed both surveys. Only four participants under 21 years old drank alcohol at baseline. Thirty participants (49.2%) were aged 21 years or older, and only eight of these participants consumed alcohol at baseline. Average alcohol use was exactly the same for both baseline and follow‐up among 14 acne cases aged 21 years or older (0.1 ± 0.1; *p* > 0.999). Among 16 controls aged 21 years or older, average alcohol use was similar between baseline (0.12 ± 0.20) and follow‐up (0.1 ± 0.2; *p* = 0.33). Specifying acne cases who used their baseline prescribed medication daily (*N* = 9) did not show a significant difference in alcohol use (baseline: 0.1 ± 0.1; follow‐up: 0.1 ± 0.1; *p* = 0.35). These averages and standard deviations were not considerably different from those found for alcohol in Tables 6 and 7.

## DISCUSSION

This exploratory study indicated that age and medication use may be related to lifestyle factors in acne patients. Skin picking improved for acne cases whose acne improved or who used their medication daily. Cases who used medication daily saw improved emotion‐related SRQL. Similarly, acne cases who used their baseline medication daily had improved anger scores. No diet‐related factors were statistically significant between baseline and follow‐up until specifying acne cases who used medication daily, where the increase on average fruit or vegetable consumption was greater. These findings indicate that (1) consistent acne medication use may be related to changes in mental/emotional health and (2) consumption of healthy foods may be related to health‐consciousness, which could also be related to consistent use of medications. However, further study with larger samples is needed to confirm whether medication use is a significant effect modifier in these relationships.

Additionally, results suggest age differences. The youngest and oldest participants often were acne‐free controls, which aligns with research showing acne is less prevalent before and after the teenage years.^38^, ^48^ Correlations with soda and milk that were similar between cases and controls suggest age could be related to certain lifestyle factors. Greater alcohol consumption was correlated with older age but was only statistically significant in acne cases. Previous research indicates significant differences in acne prevalence between alcohol drinkers and non‐drinkers with adolescent acne but not adult acne.^38^ Alcohol consumption may be related to age, but further evidence is needed to understand why an association between alcohol and acne exists.

Further, this study indicated that cross‐sectional selection of acne‐free controls could miss acne flares that develop in the near‐term after study evaluation. There was a small percentage of acne‐free controls who indicated acne improvement, suggesting they may have developed acne flares over the 6 weeks. It is possible that changes in acne medication use or lifestyle could have affected acne flares after baseline evaluation, but further detail on timing of flares and related medication or lifestyle factor frequency for these controls was not collected.

### Limitations

Reviewing literature on diet, mental health, and acne reveals dozens of observational studies, but prospective studies are less common and tend to be clinical trials, which often have relatively small sample sizes.^8^, ^13^, ^17^, ^49^, ^50^, ^51^, ^52^ Larger prospective studies are needed to better understand these relationships. One challenge, however, is retention; the current study obtained a reasonable baseline sample, but less than one‐third of baseline participants completed follow‐up surveys. The current study had no funding to incentivize participants, which might have decreased follow‐up participation; however, data on barriers related to financial incentives are mixed.^53^, ^54^, ^55^ Another limitation was finding a sufficient number of acne‐free controls in the 12‐ to 24‐year‐old age range, given that so many patients in that age range had at least mild acne. Observational studies in populations where acne is less prevalent or clinical trials that only enrol acne patients may be necessary study designs for future research on acne and lifestyle.

Furthermore, causality cannot be determined by the current study. For example, acne cases who used their baseline medication daily indicated increased fruit or vegetable consumption 6 weeks later. This could be due to chance, especially considering the relatively small sample size of the group that completed both surveys, but this could also be due to health consciousness. There may also be other lifestyle‐related variables that were not accounted for in this study but could influence results. Clinical trials are needed to confirm causality.

### Future direction

Lifestyle medicine has potential for creating complementary medical treatments for acne. Many are calling for reform and increased focus on medical nutrition education and training. Lifestyle medicine education, particularly relating to diet and nutrition, is limited in the current medical training system,^56^, ^57^ with an average of 19 total hours over 4 years and a focus primarily on vitamin deficiencies and biochemical pathways.^56^ Specialty training programs like dermatology do not require nutrition education as part of graduate requirements. However, further study is needed on how lifestyle relates to acne to inform instruction in dermatology, and lifestyle medicine could benefit from including allopathic knowledge. The current study indicates that combining knowledge of allopathic medications and lifestyle is more likely to provide sufficient evidence on lifestyle relationships.

Many studies have evaluated lifestyle factors with acne vulgaris, but few prospective observational studies have been conducted to assess change over time. This study provides evidence for changes over time that have not been captured in previous cross‐sectional studies on acne and lifestyle factors, and results indicate that medication use and patient age may be crucial to understanding true relationships between acne and lifestyle.

## AUTHOR CONTRIBUTIONS

Drs. Snyder and Secrest initially developed ideas for this project. Drs. Playdon, Murtaugh, Vanneman, Greene, and Ufkes and Ms. Stone contributed to further methods development. Drs. Snyder, Secrest, and Ufkes and Ms. Stone contributed to recruitment of participants. Dr. Greene and Dr. Snyder calculated sample size. Dr. Snyder conducted statistical analyses of data. Dr. Snyder led the writing of the initial manuscript with contributions from Dr. Ufkes and Ms. Stone. All co‐authors had a chance to revise the manuscript and contribute edits.

## CONFLICT OF INTEREST STATEMENT

Dr. Tom Greene has received research support from Boehringer‐Ingelheim, Pfizer Inc, CSL‐Behring, Novartis, Janssen Pharmaceuticals, and AstraZeneca. None of these companies had a direct association with this study. Drs. Snyder, Ufkes, Playdon, Murtaugh, Vanneman, and Secrest and Ms. Stone have no conflicts of interest to declare.

## ETHICS STATEMENT

All patients in this manuscript have given written informed consent for participation in the study and the use of their deidentified, anonymized, aggregated data and their case details for publication. This project received ethics approval from the University of Utah Institutional Review Board (#140574).

## Supporting information

Supporting information.

## Data Availability

Research data are not shared.
