# Supplementary material for: Lifestyle, medication use, and age considerations with acne vulgaris: A prospective study
Source: JEADV Clin Pract. 2024 Aug 15;4(1):82–94. doi: 10.1002/jvc2.521 (PMC12788406; doi:10.1002/jvc2.521)
Supplement: Supplementary file 1 — Supporting information. [file JVC2-4-82-s001.docx]

**Lifestyle, Medication Use, and Age Considerations with Acne Vulgaris: A Prospective Study**

Ashley M. Snyder,^1,2,3^ Caroline J. Stone,^4^ Nicole Ufkes,^1^ Tom Greene,^2,3^ Mary C. Playdon,^2,5,6^ Maureen A. Murtaugh,^2,3,5^ Megan E. Vanneman,^2,3^ Aaron M. Secrest^1,2,7^

^1^ Department of Dermatology, University of Utah, Salt Lake City, Utah, United States

^2^ Department of Population Health Sciences, University of Utah, Salt Lake City, Utah, United States

^3^ Division of Epidemiology, Department of Internal Medicine, University of Utah, Salt Lake City, Utah, United States

^4^ School of Medicine, University of Utah, Salt Lake City, Utah, United States

^5^ Department of Nutrition & Integrative Physiology, University of Utah, Salt Lake City, Utah, United States

^6^ Cancer Control and Population Sciences, Huntsman Cancer Institute, University of Utah, Salt Lake City, Utah, United States

^7^ Department of Dermatology, Christchurch Hospital, Health New Zealand Te Whatu Ora, Christchurch, New Zealand

**Corresponding Author:**

Ashley M. Snyder, PhD, MPH

Division of Epidemiology

Department of Internal Medicine

University of Utah

383 Colorow Drive, Room 344

Salt Lake City, Utah 84108, United States

E-mail: ashley.snyder@utah.edu

**Supplementary material 1.** Descriptions of survey questions for baseline and follow-up surveys

*Consent*

Participants completed electronic informed consent in the REDCap survey before they could proceed with study-related questions. An assent form and parental permission form were completed by the participant and a parent or other legal guardian if the participant was under 18 years old at baseline. A participant who was 17 at baseline but turned 18 years old before or on the day the follow-up survey was sent had to complete a consent document to proceed with the follow-up survey.

*Demographics*

Demographic questions were included at the beginning of the baseline survey only. Questions on age, sex, race, and ethnicity were from the Standard High School version of the 2021 State and Local Youth Risk Behavior Survey (YRBS).^1^ Age options were modified to reflect the age range in the current study (12-24 years). Responses for race and ethnicity were combined to compare non-Hispanic White participants to all others (including mixed race since the race question allowed multiple options to be selected).

Investigator-developed questions on school and employment were included in both the baseline and follow-up surveys. For the school question, participants were asked, “Which of the following best describes where you currently are in school?” Answer options included “Middle school,” “High school,” “College or trade school after high school,” “Graduate school,” and “No longer in school (graduated or GED).” For the employment question, participants were asked, “Which of the following best describes your current job status? **(Do not include school here.)**” Answer options included “Full-time job only,” “Part-time job(s) only,” “Both full- and part-time jobs,” and “Unemployed.”

*Diet assessment*

Dietary intake questions from the Standard High School version of the 2021 State and Local YRBS^1^ assessed the frequency of consuming select foods and beverages, including 100% fruit juice (excluding fruit-flavored drinks), fruit, green salad, potatoes (excluding French fries, fried potatoes, and potato chips), carrots, other vegetables, soda (or pop, excluding diet beverages), cow’s milk, and alcohol (excluding a few sips of wine for religious purposes). YRBS questions were assessed for reliability in early versions,^2,3^ and YRBS fruit and vegetable questions have been shown to be valid for high school students.^4^ The YRBS was developed for high school-aged adolescents (approximately ages 14 to 18 years).^2-4^

The YRBS alcohol question was amended to 1) include a description of what counts as alcohol from the alcohol drinking habits and drinking history section of the YRBS, 2) ask about consumption during the past seven days instead of the YRBS timeframe of the past 30 days, and 3) have an answer range similar to questions on fruit juice and soda. The question on milk consumption was revised to specify cow’s milk to distinguish from plant-based milks since consumption of dairy milk was positively associated with acne in prior studies.^5^

Each variable had seven answer options ranging from no consumption to consumption four or more times (or glasses for milk) over the past seven days. Food and beverage variables were converted to continuous variables assessing number of times (or glasses for milk) per day using a method based on the 2005-2006 National Health and Nutrition Examination Survey.^6^ Answers indicating no consumption were given a value of 0, and values of 1, 2, 3, and 4 were given to answers of “1 time per day,” “2 times per day,” “3 times per day,” and “4 or more times per day,” respectively. Answer options of “1 to 3 times during the past 7 days” and “4 to 6 times during the past 7 days” were given values of 0.29 and 0.71, respectively. A “fruits or vegetables” variable was created by adding values for the fruit, green salad, potatoes, carrots, and other vegetables variables.

*Mental and emotional health assessments*

The validated 25-item Connor-Davidson Resilience Scale assessed emotional resilience; scores range from 0 to 100, with higher scores indicating greater resilience.^7^ The NIH Toolbox Perceived Stress Fixed Form Age 18+ v2.0, PROMIS Short Form v1.0 - Depression 4a, PROMIS Short Form v1.0 - Anxiety 4a, PROMIS Short Form v1.1 - Anger 5a, and PROMIS Short Form v1.0 - Sleep Disturbance 4a assessed perceived stress, depression, anxiety, anger, and sleep disturbance, respectively. These measures from HealthMeasures^8^ were obtained via REDCap’s Shared Library.^9^ REDCap surveys auto-calculated T-scores. Higher scores indicated worse perceived stress, depression, anxiety, anger, or sleep disturbance.^8,10^ The mean score is 50, with a standard deviation of 10, for the original reference population for testing these measures.^10^

*Skin-related quality of life assessments*

Skin-related quality of life (SRQL) was evaluated using the validated Skindex-16, which includes three domain: symptoms, emotions, and functioning.^11^ Scores for each domain were calculated by taking the sum from each question in the domain (with values ranging from 0 to 6), multiplying this sum by 16.6667, and dividing by the total number of questions in the scores; instructions on scoring the Skindex-16 were provided by ePROVIDE Mapi Research Trust.^12^ The score range for each domain is approximately 0 to 100, and higher scores indicate worse impact on SRQL.^11^ Because this study was focused on acne but included acne-free participants, the Skindex-16 instructions for participants were modified to ask participants to reflect on their acne or other skin condition if they did not have acne at the time of the survey. This change to instructions was made with permission from ePROVIDE Mapi Research Trust.^12^

*Physical activity*

A YRBS question assessed physical activity by asking about the number of days during the past seven days when participants engaged in physical activity for at least 60 minutes per day.

*Cigarette smoking*

A question on cigarette smoking from the YRBS was modified to create a cigarette smoking question that looks at the past seven days and adds emphasis on the topic by bolding “smoke cigarettes” for consistency with other questions.

*Skin irritants*

Investigator-developed questions on skin irritants (make-up and skin picking) were included in both surveys. These were designed to be similar in appearance to the previous questions on food, physical activity, and cigarette smoking for consistency. The make-up question was turned into a binary yes/no variable for calculations.

*Medications*

Questions on medications were investigator-developed (Supplementary Table 1). Medication use questions were included in both surveys to understand how medications might be related to acne and help determine whether a participant met exclusion criteria. A medication satisfaction question was included in the baseline survey only. The follow-up survey included questions on medication adherence to assess the influence of medication use six weeks after the patient’s dermatology visit. At the start of the medication adherence section, participants were given the instructions, “Think back to the dermatology visit you had six weeks ago.” In this section, all participants received a question on acne since acne could develop within the six weeks after their visit. Some questions used branching logic.

A question about menstruation was added to the beginning of the section about medication use for both surveys. This question only appeared to participants who indicated they were female in the sex question from the baseline survey. Information on menstruation was intended to be used for understanding how hormones might be related to acne in female participants. For this question, participants were asked, “When was the last time you had your menstrual period?” Response options included “Never had one,” “I am currently on my period,” “One week ago,” “Between two to four weeks ago,” and “Five or more weeks ago.”

If a participant did not complete a baseline survey, this participant was to be excluded from primary analyses because baseline medication use questions helped confirm accuracy of electronic medical record (EMR) reporting of acne-related medications^13^ used within the past seven days. Acne-related medications (except hormonal contraceptives) did not have to be indicated for acne in the EMR or recruiter notes for inclusion. Moisturizers for acne-related dryness and medications applied during study-related dermatology visits were not included as acne-related medications.

References

1. 2021 Youth Risk Behavior Survey Questionnaire. Centers for Disease Control and Prevention. Available at: [www.cdc.gov/yrbs](file:///C:\Users\Ashley%20Snyder\Desktop\F31%20Grant%20Application%202020\www.cdc.gov\yrbs) (last accessed 28 February 2023).

2. Brener ND, Collins JL, Kann L, Warren CW, Williams BI. Reliability of the Youth Risk Behavior Survey questionnaire. *Am J Epidemiol*. 1995;**141**(6):575-80.

3. Brener ND, Kann L, McManus T, Kinchen SA, Sundberg EC, Ross JG. Reliability of the 1999 Youth Risk Behavior Survey questionnaire. *J Adolesc Health*. 2002;**31**(4):336-42.

4. Eaton DK, Olsen EO, Brener ND, Scanlon KS, Kim SA, Demissie Z, et al. A comparison of fruit and vegetable intake estimates from three survey question sets to estimates from 24-hour dietary recall interviews. *J Acad Nutr Diet*. 2013;**113**(9):1165-74.

5. Aghasi M, Golzarand M, Shab-Bidar S, Aminianfar A, Omidian M, Taheri F. Dairy intake and acne development: a meta-analysis of observational studies. *Clin Nutr*. 2019;**38**(3):1067-75.

6. National Center for Health Statistics. Food Frequency Questionnaire - Output from DietCalc Software (FFQDC_D). Centers for Disease Control and Prevention. 2008. Available at: <https://wwwn.cdc.gov/Nchs/Nhanes/2005-2006/FFQDC_D.htm#:~:text=A%20food%20frequency%20questionnaire%20%28FFQ%29%20%28sometimes%20referred%20to,months.%20Two%20public%20data%20release%20files%20were%20prepared> (last accessed 3 May 2024).

7. Connor KM, Davidson JR. Development of a new resilience scale: the Connor-Davidson Resilience Scale (CD-RISC). *Depress Anxiety*. 2003;**18**(2):76-82.

8. Search & View Measures. HealthMeasures. Available at: <https://www.healthmeasures.net/search-view-measures> (last accessed 12 February 2023).

9. Obeid JS, McGraw CA, Minor BL, Conde JG, Pawluk R, Lin M, et al. Procurement of shared data instruments for research electronic data capture (REDCap). *J Biomed Inform*. 2013;**46**(2):259-65.

10. Interpret Scores. HealthMeasures. Available at: <https://www.healthmeasures.net/score-and-interpret/interpret-scores> (last accessed 12 February 2023).

11. Chren MM, Lasek RJ, Sahay AP, Sands LP. Measurement properties of Skindex-16: a brief quality-of-life measure for patients with skin diseases. *J Cutan Med Surg*. 2001;**5**(2):105-10.

12. Mapi Research Trust. Official Skindex, Skindex-29, Skindex-16 distributed by Mapi Research Trust. ePROVIDE. Available at: <https://eprovide.mapi-trust.org/instruments/skindex> (updated February 2023; last accessed 12 February 2023).

13. Graber E. Acne vulgaris: Overview of management. UpToDate. Available at: [https://www.uptodate.com/contents/acne-vulgaris-overview-of-management#](https://www.uptodate.com/contents/acne-vulgaris-overview-of-management)! (updated 23 February 2023; last accessed 3 May 2024).

**Supplementary Table 1.** Medication questions for baseline and follow-up surveys addressing medication use, satisfaction, and adherence

| **Category** | **Question** | **Response options** | **Branching logic** |
| --- | --- | --- | --- |
| Use | In the past 7 days, have you used any topicals on your skin (for example, creams, acne face washes, face pads/wipes, patches)? | Yes, No | None |
|  | In the past 7 days, did you use any non-topical **prescription** medications (such as pills, injections, or liquid medicines)? | Yes, No | None |
|  | Have you used hormonal contraceptives in the form of pills, patch, implant, IUD, or vaginal ring at some point within the past month **or** in the form of an injection at some point within the past three months? | Yes, No | If “Female” in baseline question on sex |
|  | Have you used **isotretinoin (also called Accutane)** at any time in your life? (This would have been pills you were supposed to take for several months and you would have had to sign up for iPledge.) | Yes, No | None |
| Satisfaction | How satified are you with the acne treatments (or treatments for another skin condition if you do not have acne) you were using before your dermatology visit today? | Very dissatisfied, Somewhat dissatisfied, Neither satisfied nor dissatisfied, Somewhat satisfied, Very satisfied | None |
| Adherence | Did you receive a medication (for example, cream, face wash, pills, or other medication) for your acne (or other skin condition if you did not have acne) at your dermatology visit? | Yes, No | None |
|  | In the past six weeks since your dermatology visit, how often, on average, have you used the medication(s) you received? | Never, Less than once a week, Once a week, Between two to seven days each week, Every day | If “Yes” to previous question about receiving a medication |
|  | In the past six weeks since your dermatology visit, how satisfied have you been with how the medication(s) from your visit have been improving your acne (or other skin condition if you did not have acne at your visit)? | Very dissatisfied, Somewhat dissatisfied, Neither satisfied nor dissatisfied, Somewhat satisfied, Very satisfied | If any answer, except “Never,” in the previous question on using medications received |
|  | In the past six weeks, have you used any medications for your acne (or other skin condition if you did not have acne at your visit) other than the one(s) you were prescribed at your University of Utah dermatology visit six weeks ago? | Yes, No | None |
|  | In the past six weeks, how often, on average, did you use these other medication(s)? | Less than once a week, Once a week, Between two to seven days each week, Every day | If “Yes” to previous question about other medications |
|  | In the past six weeks, how satisfied have you been with these other medication(s) helping your acne (or other skin condition if you did not have acne at your visit)? | Very dissatisfied, Somewhat dissatisfied, Neither satisfied nor dissatisfied, Somewhat satisfied, Very satisfied | If “Yes” to previous question about other medications |
|  | Thinking back to your dermatology visit six weeks ago, which of the following best describes the severity of your acne since that visit? **(If you were not seen for acne at your visit six weeks ago and have not had acne since, please mark "Not applicable".)** | Not applicable, Acne improved, Acne stayed the same, Acne got worse | None |
|  | Compared to how you felt six weeks ago, how satisfied overall are you with the treatments you are using for your acne (or other skin condition if you did not have acne six weeks ago)? | Much more dissatisfied than six weeks ago, Somewhat more dissatisfied than six weeks ago, Neither more satisfied nor dissatisfied than six weeks ago, Somewhat more satisfied than six weeks ago, Much more satisfied than six weeks ago | None |

**Supplementary Table 2.** Definitions for the two Investigator’s Global Assessments for acne

| **Five-category Investigator’s Global Assessment** | | | |
| --- | --- | --- | --- |
| **Score** | **Severity** | | **Evaluation criteria** |
| 0 | Clear | | Residual hyperpigmentation and erythema may be present. |
| 1 | Almost clear | | A few scattered comedones and a few small pustules. |
| 2 | Mild | | Easily recognizable; <1/2 face is involved. Some comedones, some papules and pustules. |
| 3 | Moderate | | >1/2 face is involved. Many comedones, papules and pustules. One nodule may be present. |
| 4 | Severe | | Entire face is involved, covered with comedones, numerous papules and pustules, and few nodules and cysts. |
| **Six-category Investigator’s Global Assessment** | | | |
| **Score** | | **Evaluation criteria** | |
| 0 | | Clear: Residual pigmentation and erythema may be seen. | |
| 1 | | A few scattered open or closed comedones and very few papules. | |
| 2 | | Easily recognizable: less than half of the face/area is involved. A few open or closed comedones and a few papules and pustules. | |
| 3 | | More than half of the face/area is involved. Many papules and pustules, many open or closed comedones. One nodule may be present. | |
| 4 | | Entire face/area is involved, covered with many papules and pustules, open or closed comedones and rare nodules. | |
| 5 | | Highly inflammatory acne covering the face/area with presence of nodules. | |
